# FRAP analysis of chromatin looseness in mouse zygotes that allows full-term development

**DOI:** 10.1371/journal.pone.0178255

**Published:** 2017-05-22

**Authors:** Masatoshi Ooga, Teruhiko Wakayama

**Affiliations:** 1 Advanced Biotechnology Center, University of Yamanashi, Kofu-shi, Yamanashi, Japan; 2 Faculty of Life and Environmental Science, University of Yamanashi, Kofu-shi, Yamanashi, Japan; Institute of Zoology Chinese Academy of Sciences, CHINA

## Abstract

Chromatin looseness, which can be analyzed by fluorescence recovery after photobleaching (FRAP) using eGFP-tagged core histone proteins, is an important index of the differentiation potential of blastomere cells and embryonic stem cells. Whether chromatin looseness is a reliable index of the developmental potential of embryos during ontogenesis is not known. As a necessary first step toward answering this question, we investigated whether FRAP-analyzed embryos are capable of normal preimplantation and full-term development. All tested concentrations (50, 100, and 250 ng/μL) of microinjected eGFP-H2B mRNA were sufficient for detecting differences in chromatin looseness between male and female pronuclei. After FRAP analysis, most of the zygotes developed into blastocysts. Importantly, a considerable number of offspring developed from the FRAP analyzed zygotes (32/78; 41.0%) and grew into healthy adults. The offspring of zygotes injected with 250 ng/μL of eGFP-H2B mRNA and bleached using 110 μW laser power for 5 s were not genetically modified. Interestingly, bleaching using a 3-fold stronger laser intensity for a 6-fold longer time did not cause toxicity during preimplantation development, indicating that bleaching did not critically affect preimplantation development. Finally, we confirmed that similar results were obtained using two different types of confocal laser-scanning microscopes. This FRAP protocol would be useful for investigating the association between chromatin looseness and development.

## Introduction

Ontogenesis begins from zygotes in which dynamic changes in the chromatin structure occur. Extensive alterations of epigenetic histone modifications and substitutions of histone variants in zygotic chromatin are thought to be important for subsequent development [1–6]. However, the biological roles and significance of these phenomena are still largely unknown.

Euchromatin-enriched open chromatin has emerged as a new epigenetic factor and is extensively studied because of its association with cellular potency. Specifically, cells with greater differentiation potential have more open chromatin; upon cellular differentiation, this open chromatin structure changes to a closed state [7–9]. A higher mobility of histone proteins and pervasive transcription are thought to be indirect indices of open chromatin structure [8]. The customary method used to observe open chromatin structure involves immuno-staining with antibodies against histone modifications. Fluorescence recovery after photobleaching (FRAP) is a new technique for such studies that uses fluorescent labeling of histones [10, 11]. This method was used to study the mobility of histones within the chromatin of pluripotent embryonic stem (ES) cells, revealing that histone proteins in open chromatin are more loosely bound and thus have higher mobility within the chromatin [11]. After differentiation from a pluripotent state into neuron precursor cells (NPC), the histones bind more tightly to the chromatin, decreasing their mobility. As transcription occurs in regions of open chromatin, the higher histone mobility in ES cells is thought to enable genome-wide transcription [8, 12]. Open chromatin structure is also observed in preimplantation embryos. We previously reported that zygotes have an extensively loosened chromatin structure that is gradually lost during preimplantation development [13]. Other studies have reported similar observations [10, 14]. In addition, pervasive transcription is gradually lost during this period of development [15], mirroring the changes in chromatin looseness during ES-cell differentiation. Accordingly, the extensively loosened chromatin structure in zygotes likely plays a role in pre- and post-implantation development, but this relationship has not been confirmed.

Because the immunostaining method used to analyze chromatin structure requires cell fixation, developmental events cannot be followed in a single embryo using this technique. However, the chromatin structure in preimplantation embryos can be analyzed by live-cell imaging without killing the cells. This technique thus allows us to investigate the link between a molecular event and subsequent development within the same embryo. In addition, by transferring the embryos into a recipient pseudopregnant female, we can obtain offspring from the observed embryos [16]. By using such an approach, we can investigate whether there is a correlation between the successes of the interested molecular events and embryonic developmental competency. Namely, we can elucidate whether our interested molecular phenomena is actually important for ontogenesis. Although live imaging analysis of pervasive transcription is impossible, FRAP can be used to monitor chromatin looseness via fluorescently-labeled histones. Therefore, by following the subsequent development, the correlation between zygotic open chromatin structure and subsequent development can be investigated. Such studies would be most informative if the zygotes were able to develop to term while under FRAP analysis. To the best of our knowledge, the viability of full-term FRAP-analyzed zygotes has not been reported. This study aims to establish an experimental system by which the open chromatin structure of zygotes can be evaluated without inflicting critical damage on the zygotes, allowing them to develop to term.

## Materials and methods

### Animals

B6D2F1 (C57BL/6 × DBA2) and ICR mice were used as oocyte and spermatozoa donors, respectively. Mice were purchased from SLC (Shizuoka, Japan). Surrogate pseudo-pregnant female mice were ICR-strain mated with vasectomized same-strain male mice. On the day of the experiments, or after having finished all experiments, the mice were euthanized by CO_2_ inhalation or by cervical dislocation. All animal experiments conformed to the Guide for the Care and Use of Laboratory Animal Experimentation of the University of Yamanashi.

### Ethics statement

This study was performed in strict accordance with the recommendations in the Guide for the Care and Use of Laboratory Animals of University of Yamanashi. The protocol was approved by the Committee on the Ethics of Animal Experiments of the University of Yamanashi (Permit Number: A24-50). All surgery was performed under tribromoethanol anesthesia, and all efforts were made to minimize suffering.

### Collection of oocytes

The unfertilized oocytes were collected from 8–12-week-old female mice induced to super-ovulate by the injection of 7.5 IU equine chorionic gonadtropin (eCG; ASKA Pharmaceutical, Tokyo) and human chorionic gonadtropin (hCG; ASKA Pharmaceutical) at 46–50 h intervals. Cumulus oocyte complexes (COCs) were obtained from the oviducts about 16 h after hCG injection. The obtained oocytes were subjected to *in vitro* fertilization (see below).

### In vitro fertilization

Spermatozoa were obtained from mature male ICR mice and then capacitated by incubation in HTF [17] for 1 h. The oocytes were then inseminated with the capacitated sperm. At 1 h post insemination, the cells were treated with hyaluronidase for 10 min in a humidified atmosphere of 5% CO_2_/95% air at 38 C. The cells were then subjected to microinjection of mRNA for FRAP analysis.

### Synthesis of mRNA

pTOPO-eGFP-H2B, constructed in a previous study [13], was linearized with *Not* I and then purified by phenol–chloroform extraction. After purification, the DNA fragment was used as the template for *in vitro transcription* with using mMESSAGE MACHINE sp6 kit (Life Technologies, Carlsbad, CA, USA). As a result, eGFP-H2B non–poly-A-tailed RNA was obtained. The mRNA was then poly-A tailed using a poly-A tailing kit (Life Technologies). The mRNA then was precipitated with lithium chloride precipitation solution from the kit and dissolved in nuclease-free water at 500 ng/μL and stored −80 C until use.

### Microinjection of mRNA

mRNA was diluted with nuclease-free water to 50, 100, 250, and 500 ng/μL before use. mRNA was injected into the cytoplasm of 1-cell-stage embryos at 1 h post-insemination using borosilicate glass capillaries (Sutter Instruments, Novato, CA, USA) as described previously [18]. Briefly, microinjection was performed in HEPES-buffered CZB [19] on an inverted microscope (Olympus, Tokyo, Japan) with a micromanipulator (Narishige, Tokyo, Japan). The zona pelucida and cytosolic membrane were penetrated with a piezo drive (PRIME Tech, Tokyo, Japan). Ten min after microinjection, the embryos were washed and cultured in CZB.

### Fluorescence recovery after photobleaching (FRAP)

The embryos were transferred into HEPES-buffered CZB covered with mineral oil. The stage of the confocal microscope was warmed with a thermo plate (Tokai Hit, Shizuoka, Japan). The region of interest (ROI), reference region (ref) and background (back) were set using Flowview software (Olympus). The areas of these regions were set at 40 × 40 pixels (7.6 μm^2^). Three pictures were taken at 1.6 s intervals (free run setting), after which the ROI was photobleached with the 110 μW laser at 488 (FV1000) or 477 (FV1200) nm for 5 s. A total of 9 pictures was taken at 1.6-s intervals, and the intensities of the ROI, ref, and back were measured in each picture. To reduce the toxicity to zygotes, the observation time (bleaching and imaging time) was set at 23 s, which is shorter than that used in our previous study (150 s) [13]. For measuring intensity, the 488 or 477-nm laser output was set at 15 μW. The recovery rate and mobile fraction were calculated as described previously [13].

### Embryo transfer

Adult female ICR mice were mated with vasectomized ICR male mice the night before transfer. Embryos at the 2-cell stage were transferred into the oviducts of pseudopregnant female ICR mice at 0.5 days post coitum (dpc). At 18.5 dpc, the offspring were recovered by cesarean section. Some were delivered to foster mothers, and the weights of these pups were assessed each week.

### Genotyping

The tails of mice were cut and then lysed with lysis buffer (0.1 M Tris pH 8.0, HCl, 0.2 M NaCl, 5 mM EDTA, 0.1% SDS) containing proteinase K at 55 C for several h. The extracted genomic DNA was purified with phenol/chloroform and then precipitated with ethanol. The obtained genomic DNA was resolved in nuclease-free water and then was used for genotyping PCR with Ex Taq DNA polymerase (Takara, Shiga, Japan). The primers used were as follows: GFP, 5′- CCTACGGCGTGCAGTGCTTCAGC-3′ (forward) and 5′-CGGCGAGCTGCACGCTGCCGTCCTC-3′ (reverse), *hist2h2bb* (H2B) 5′- ATCACTTCCCGGGAGATCCA-3′ (forward) and 5′- AGCCTTTTGGGTAAAGCCGA-3′ (reverse). A GFP-expressing transgenic ICR mouse and a mouse derived from intact were used as a positive and negative control, respectively. PCR was performed by denaturation at 95 C for 2 min followed by 30 cycles at 95 C for 30 s (denaturation), 65 C for 30 s (annealing), and 72 C for 30 s (elongation). PCR products were separated on 2% agarose gels and stained with ethidium bromide. The gel image was obtained using a STAGE 2000 UV illuminator (VILBER LOURMAT, Collegien, France).

### Statistical analysis

The birth rate of pups was evaluated by the chi-square test using Prism7 software (Graphpad, La Jolla, CA, USA). *P* < 0.05 was considered a significant difference.

## Results

### Effect of FRAP observation on preimplantation development

**Fig 1:**
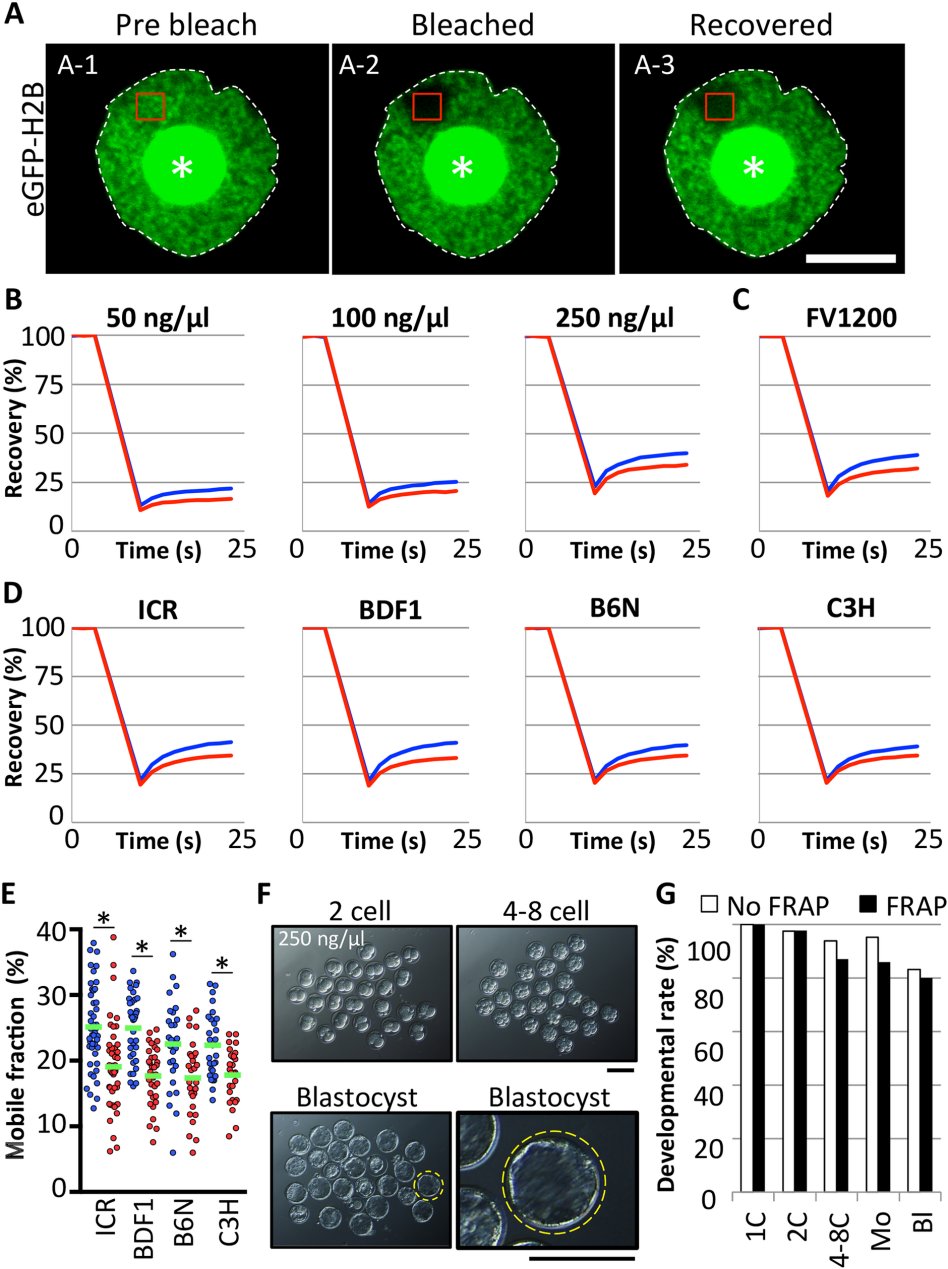
Optimization of fluorescence recovery after photobleaching (FRAP). (A) mRNA encoding eGFP-H2B was injected into the cytoplasm of 1-cell-stage embryos 1 h post-insemination (hpi). The eGFP-H2B–expressing zygotes were collected at 8–12 hpi and subjected to FRAP analysis. Representative photographs of FRAP analysis were taken before bleaching (A-1), soon after bleaching (A-2), and after recovery (A-3). Regions of interests (ROIs) are indicated by red rectangles. The nuclear membranes (white dotted lines) and nucleoli (asterisks) are indicated. Scale bar = 10 μm. (B) Recovery curves obtained from zygotes injected with eGFP-H2B mRNA at 50, 100, and 250 ng/μL. Curves indicate fluorescence recovery in male (blue) and female (red) pronuclei. Three independent experiments were performed in which 31, 30, and 21 embryos were examined in total, respectively. (C) Recovery curve obtained using newer confocal laser scanning microscope (FV1200). Data shown are from three independent experiments, examining 58 embryos in total. (D, E) Recovery curves and mobile fraction obtained from zygotes prepared by using spermatozoa from ICR, BDF1, B6N, and C3H. At least three experiments were performed in which 45, 35, 27 and 31 embryos were used, respectively. Single dots indicate individual pronuclei; blue and red dots indicate the mobile fraction score obtained from each male and female pronuclei, respectively. Asterisks indicate significant differences between male and female pronuclei (*P* < 0.05, by paired *t* test) (F) Preimplantation development of FRAP-analyzed embryos with eGFP-H2B mRNA at 250 ng/μL. Representative images of FRAP-analyzed preimplantation developing embryos are shown. Two-cell, 4–8 cell, and blastocyst stage embryos at 24, 48, and 96 hpi, respectively. Yellow circle indicates the well-developed blastocyst. This blastocyst is enlarged and shown on the right panel. Scale bar = 100 μm. (G) Bar graph of developmental rates of FRAP-analyzed embryos. Bars indicate FRAP-analyzed (FRAP) embryos (black) and controls embryos (white) injected with mRNA but no FRAP analysis (No FRAP). Data shown are from three independent experiments, examining at least 57 embryos in total. Two-cell, 4–8 cell, morula (Mo), and blastocyst (Bl) stages were observed at 24, 48, 72, and 96 hpi, respectively.

**Fig 2:**
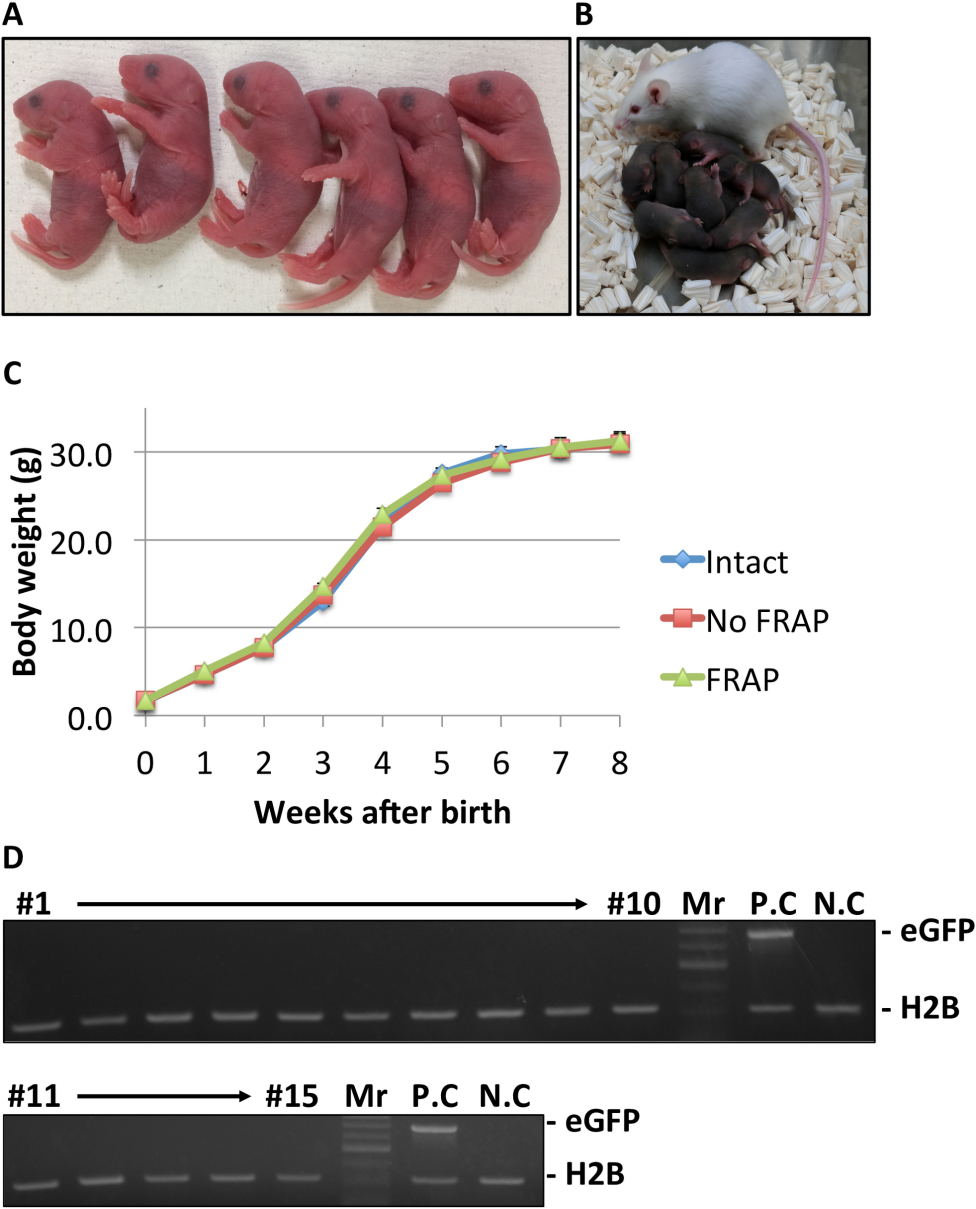
Healthy pups derived from FRAP-analyzed zygotes. Photographs of the pups obtained from FRAP-analyzed zygotes (A). Normal growth was observed during nursing of the FRAP-analyzed pups (B) and chart indicating the weights of pups derived from intact embryos (blue), unbleached control embryos (red), and FRAP-analyzed embryos (green). Body weights of 29 pups derived from intact embryos, 24 from no-FRAP embryos, and 15 from FRAP-analyzed embryos over an 8-week period. (D) PCR genotyping of 15 pups derived from FRAP-analyzed embryos. DNA was extracted from tails and purified for use as PCR templates. Mr, indicates molecular marker. P.C (positive control) was prepared with using a GFP-expressing transgenic ICR mouse. A mouse derived from intact zygotes was used as an N.C (negative control).

**Fig 3:**
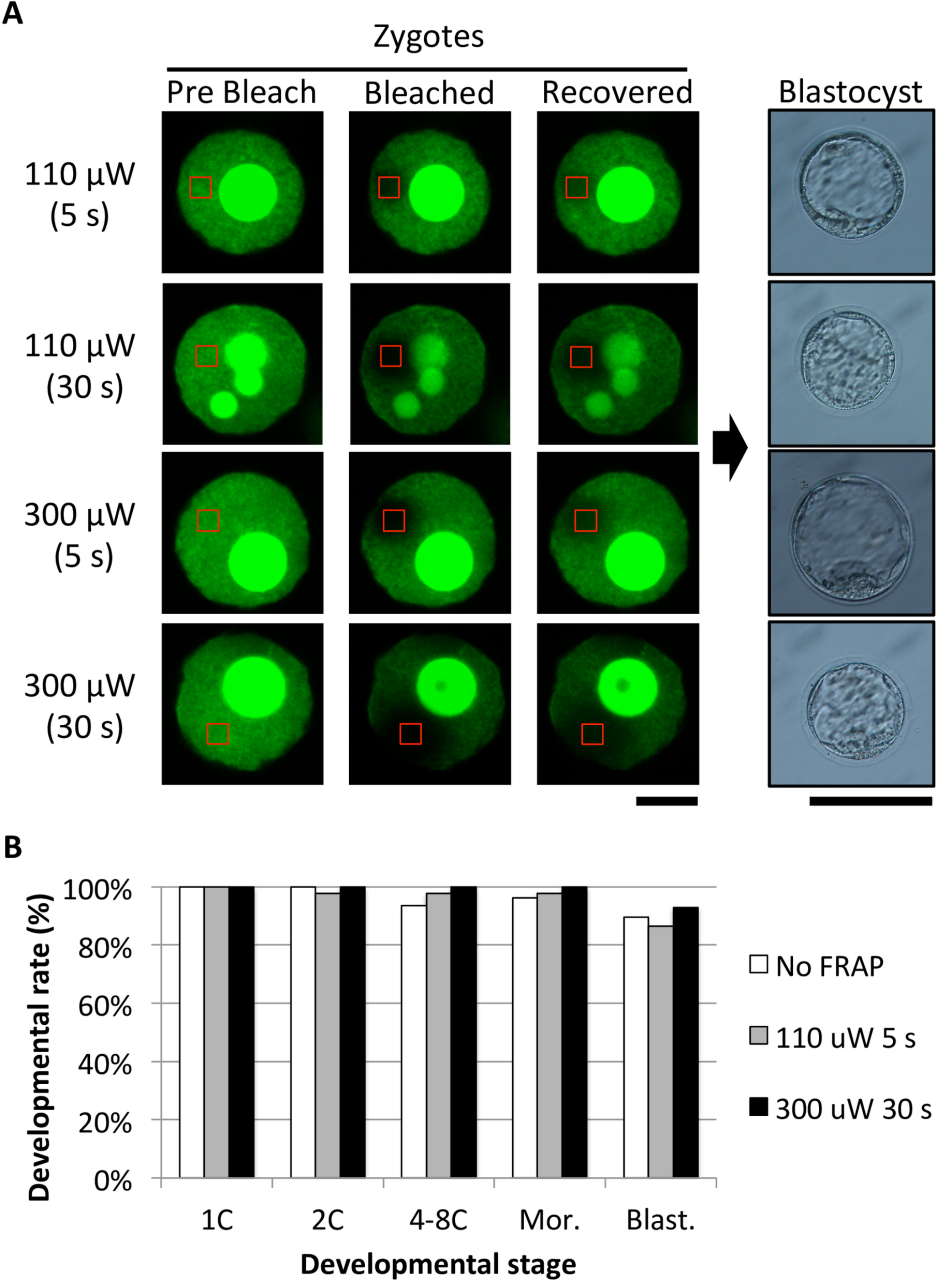
Tolerance of zygotes to photobleaching. (A) The effects of stronger and/or longer photobleaching on preimplantation development were investigated. Preimplantation development of the embryos subjected to 3-fold stronger (300 μW) and/or 6-fold longer (30 s) bleaching than the standard conditions (110 μW; 5 s). Confocal images of embryos during FRAP analysis. ROIs are indicated by red rectangles. Scale bar = 10 μm. The blastocysts shown on the right were derived from the FRAP-analyzed zygotes shown on the left. (Scale bar = 100 μm). (B) Rate of development of embryos subjected to standard (110 μW, 5 s) and stronger and longer (300 μW, 30 s) bleaching: unbleached control, white; standard, gray; and stronger/longer bleaching, black. Three independent experiments were performed in which at least 42 embryos were examined in total. Two-cell, 4–8 cell, morula (Mor), and blastocyst (Blast) stages were observed at 24, 48, 72, and 96 hpi, respectively. There is no significant difference between 110 μW, 5 s and 300 μW, 30 s; *P* value by Fisher’s exact test is *P* < 0.9999 (2C-Mor.) and *P* = 0.7136 (Blast.).

For FRAP experiments to analyze chromatin looseness, the fluorescent histone eGFP-H2B was expressed in zygotes by mRNA injection. First, we performed FRAP to elucidate the optimal concentration of mRNA for analysis. The FRAP experiment consists of bleaching and recovery steps. During bleaching, the region of interest (ROI) (Fig 1A-1) is exposed to a very strong laser; the fluorescent signal then decreases to a negligible level leaving it “bleached” as compared to the surrounding area (Fig 1A-2). After bleaching, the fluorescent signal gradually increases during the recovery phase (Fig 1A-3) because of the inflow of unbleached eGFP-H2B into the ROI. We previously demonstrated that the chromatin structure is looser in paternal pronuclei than in maternal pronuclei [13]. Thus, in this study, we used this parental difference as an evaluation criterion for determining the success of FRAP experiments. The parental difference in chromatin looseness was observable at all concentrations tested (Fig 1B). A sharper fluorescence recovery of eGFP-H2B was observed only in zygotes injected with mRNA at 250 ng/μL. Similar results were obtained using a confocal laser-scanning microscope (FV1000) and another microscope (FV1200), indicating very high reproducibility of the FRAP experiment (Fig 1C). The parental asymmetry of chromatin looseness was also observed from the zygotes prepared using spermatozoa from various mice strains; the parental asymmetry of chromatin looseness was conserved in mice (Fig 1D and 1E). Most of the FRAP-analyzed embryos developed to the blastocyst stage, at a level comparable to that of non-bleached embryos (Fig 1F and 1G; *P* = 0.7477). If live pups developed from zygotes injected with higher concentrations of mRNA, it is likely that live pups would also develop from zygotes injected with lower concentrations. Indeed, although there are no significant difference compared to intact control and most of injected zygote could develop to blastocyst, developmental rates to the blastocysts were slightly decreased with increases of the mRNA concentration (Table 1). Therefore, we decided to examine whether zygotes injected with 250 ng/μL mRNA could develop into full-term pups.

**Table 1:** Preimplantation development of the zygotes injected with mRNA at various concentrations.

Concentration of mRNA	1 cell (%) (12 hpi)	2 cell (%) (24 hpi)	4–8 cell (%) (48 hpi)	Mor. (%) (72 hpi)	Blast. (%) (96 hpi)
Intact	72 (100)	70 (97.2)	66 (91.7)	68 (94.4)*	66 (91.7)
50	63 (100)	63 (100)	62 (98.4)	61 (96.8)	57 (90.5)
100	70 (100)	70 (100)	67 (95.7)	67 (95.7)	63 (90.0)
250	72 (100)	71 (98.6)	64 (88.9)	64 (88.9)	58 (80.6)
500	71 (100)	71 (100)	67 (94.4)	66 (93.0)	59 (83.1)

*: At 72 hpi, the embryos developed to morula stage

### Full-term development of FRAP-analyzed zygotes

We investigated the development of zygotes that had been analyzed by FRAP. Embryos injected with mRNA but not bleached were prepared as controls. The first cleavage was not inhibited by bleaching and recovery (Table 2). FRAP-analyzed 2-cell stage embryos were transferred into the oviducts of pseudopregnant mothers. The birth rate of FRAP-analyzed embryos (41.6%) was slightly but significantly lower than that of intact embryos (62.2%) but the same as that of no FRAP controls (52.6%) (Table 2). Thus, FRAP analysis was slightly detrimental to full-term embryonic development. Regardless of the slight reduction in birth rate, the pups derived from both FRAP-analyzed and unbleached eGFP-H2B-expressing zygotes looked healthy and fully developed (Fig 2A, 2B, and 2C). To confirm whether mRNA injection caused genetic modifications resulting in transgenic offspring, we genotyped offspring derived from FRAP-analyzed zygotes. As shown in Fig 2D, none of the offspring demonstrated genetic modifications (Table 2).

**Table 2:** Birth rate of analyzed embryos.

Categories	No. of injected zygotes	No. of recovered zygotes after mRNA injection (%)*	No. of zygotes analyzed	No. of transferred 2-cell stage embryos (%)**	No. of recipients	No. of pups (%)***	Weight of pups (g)	No. of transgenic pups
Intact	-	-	99	98 (99.0)	9	61 (62.2)^a^	1.64±0.02	n.d
No FRAP	420	398 (94.8)	82	78 (95.1)	7	41 (52.6)^a, b^	1.66±0.03	n.d
FRAP			79	78 (98.7)	7	32 (41.0)^b^	1.69±0.03	0

*: calculated by dividing with no. of injected zygotes

**: calculated by dividing with no. of zygotes analyzed

***: calculated by dividing with no. of transferred 2-cell stage embryos. a,b: superscripts indicate significant difference (P<0.05). n.d: not determined

### Resistance of zygotes to photobleaching

Finally, we examined the resistance of zygotes to photobleaching. Zygotes were bleached using the standard method (110 μW laser power for 5 s), stronger power (300 μW laser power), or longer time (30 s), and their potential to develop to the blastocyst stage was compared. As expected, the stronger and/or longer bleaching resulted in the disappearance of the fluorescent signal in a larger area than that of zygotes treated under standard conditions (Fig 3A). Notably, bleaching with the 300 μW laser for 30 s caused much larger bleached areas that corresponds to nearly half of the nucleus (Fig 3A, left). Analysis of embryonic development revealed that even these thoroughly bleached embryos could develop to the blastocyst stage (Fig 3A, right). As shown in the bar graph, no obvious toxicity affecting preimplantation development was observed with stronger and longer bleaching as compared to bleaching at 110 μW for 5 s.

## Discussion

In this study, we examined whether FRAP analysis impairs embryo development. When FRAP analysis was performed using injected eGFP-H2B mRNA (250 ng/μL) and bleaching with 110 μW laser power for 5 seconds, clear results were obtained without any significant damage to the embryos (Fig 1F and 1G) (Table 2). After embryo transfer, many healthy offspring were obtained (Fig 2A, 2B and 2C) (Table 2). In addition, stronger and longer bleaching caused a larger bleached area but did not impair preimplantation development (Fig 3A and 3B). Because healthy pups were obtained from FRAP-analyzed zygotes, our experimental system can be used to study the association between zygotic open chromatin structure and pre- and post-implantation development.

Previous studies reported that FRAP using a 280 μW laser at 7.5 min intervals (56 000 pictures taken in total) did not critically affect the birth rate of pups [16]. Moreover, Terashita and colleagues reported that even when oocytes were exposure to 100 mW of mercury light for 5 minutes, they did not lose their full-term developmental potential after fertilization [20]. In this study, the bleaching laser power was 110 μW, and the irradiated area of the zygote for bleaching was limited to a small ROI inside the nucleus. Indeed, stronger and longer bleaching (300 μW for 30 s) did not severely hamper preimplantation development (Fig 3B). Moreover, live pups developed from the zygotes bleached using 920 μW laser power for 30 s (data not shown). These results are not surprising when compared to those of previous reports. Thus, FRAP analysis seems not to be critically detrimental to embryonic development. There may be room for improvement in the birth rates of FRAP-analyzed zygotes.

In this study, the bleaching phase was only 5 s, and the recovery phase was less than 20 s; these times are shorter than those of previous studies (60–150 s [10, 13]). By shortening the bleaching and recovery phases, we were able to analyze more zygotes in a fixed time. In addition, the shortened observation phase allowed for the culturing of zygotes without using any expensive culture systems attached to a confocal microscope system. For our FRAP analysis, only a thermo plate with a hole was needed for use with the confocal laser scanning microscope, but it did not cause any damage to the zygote because healthy pups developed from the zygotes, even those that were cultured simply in warm HEPES-buffered medium. We conclude that about 25 s of imaging time is sufficient and efficient for the analysis of chromatin looseness in many zygotes.

The synergistic effect of eGFP-H2B expression, the mRNA injection itself, and FRAP analysis slightly but significantly reduced the birth rate of pups (Table 2). Since the expression of eGFP-H2B is required for FRAP analysis, it is not possible to completely eliminate the detrimental effects of ectopic expression of this protein. However, in this study, we decided to use a high concentration of mRNA to determine whether the mRNA injection is detrimental. Although the birth rate of FRAP-analyzed embryos was slightly lower than that of intact controls, the effectiveness of FRAP analysis was not affected, even at lower concentrations of injected mRNA (Fig 1). Therefore, use of the minimum-required amount of eGFP-H2B may contribute to obtaining live, healthy pups from FRAP-analyzed zygotes. In addition, eGFP-H2B transgenic mice may become available, avoiding the physical damage caused by mRNA injections. The use of such transgenic mice might allow for a higher yield of pups after FRAP analysis.
